# Cochlear implantation trough the middle cranial fossa: a novel approach to access the basal turn of the cochlea

**DOI:** 10.5935/1808-8694.20130028

**Published:** 2015-11-02

**Authors:** Aline Gomes Bittencourt, Robinson Koji Tsuji, João Paulo Ratto Tempestini, Alfredo Luiz Jacomo, Ricardo Ferreira Bento, Rubens de Brito

**Affiliations:** a Doctoral student in the Department of Otorhinolaryngology, Medical School, University of São Paulo, São Paulo, Brazil (Fellow in Otological and Skull Base Surgery at Hospital das Clínicas, University of São Paulo, São Paulo, Brazil); b PhD in Sciences (Associate Physician in the Department of Otorhinolaryngology, Hospital das Clínicas, University of São Paulo, São Paulo, Brazil); c Medical student (Medical student, Medical School, University of São Paulo, São Paulo, Brazil); d Associate Professor (Professor in the Human Structural Topography Course at the Department of Surgery, Medical School, University of São Paulo, São Paulo, Brazil); e Associate Professor (Professor in the Otorhinolaryngology Course, Medical School, University of São Paulo, São Paulo, Brazil); f Associate Professor (Associate Professor in the Otorhinolaryngology Course, Medical School, University of São Paulo, São Paulo, Brazil). Federal University of São Paulo, São Paulo, Brazil

**Keywords:** cochlear implantation, cranial fossa, middle, deafness, hearing loss, sensorineural, neuroanatomy

## Abstract

The classic approach for cochlear implant surgery includes mastoidectomy and posterior tympanotomy. The middle cranial fossa approach is a proven alternative, but it has been used only sporadically and inconsistently in cochlear implantation.

**Objective:**

To describe a new approach to expose the basal turn of the cochlea in cochlear implant surgery through the middle cranial fossa.

**Method:**

Fifty temporal bones were dissected in this anatomic study of the temporal bone. Cochleostomies were performed through the middle cranial fossa approach in the most superficial portion of the basal turn of the cochlea, using the meatal plane and the superior petrous sinus as landmarks. The lateral wall of the internal acoustic canal was dissected after the petrous apex had been drilled and stripped. The dissected wall of the inner acoustic canal was followed longitudinally to the cochleostomy.

**Results:**

Only the superficial portion of the basal turn of the cochlea was opened in the fifty temporal bones included in this study. The exposure of the basal turn of the cochlea allowed the visualization of the scala tympani and the scala vestibuli, which enabled the array to be easily inserted through the scala tympani.

**Conclusion:**

The proposed approach is simple to use and provides sufficient exposure of the basal turn of the cochlea.

## INTRODUCTION

The classic approach for cochlear implant (CI) surgery includes mastoidectomy and posterior tympanotomy^1^. On occasion, modified approaches are required to overcome surgical peculiarities and allow the safe placement of the CI^1^, ^2^, ^3^, ^4^, ^5^, ^6^, ^7^, ^8^, ^9^, ^10^, ^11^.

The middle cranial fossa (MCF) approach is a proven, valuable approach, although it has been used only sporadically in CI surgery without much procedural standardization to handle cases with ossified cochleae, chronic suppurative otitis media, or inner ear dysplasia^2^, ^3^, ^5^, ^9^, ^12^, ^13^, ^14^, ^15^. Additionally, the lack of well-defined landmarks based on the temporal bone and the significant variability of anatomic parameters among individuals render this approach as one of the most difficult of the skull base procedures, even when performed by highly skilled surgeons^16^, ^17^, ^18^, ^19^, ^20^. This approach may also be associated with severe complications such as injuries to the facial nerve and cerebral and vascular structures^3^, ^14^, ^17^, ^18^, ^20^, ^21^.

The anatomy of the human temporal bone is regarded as highly complex, with nerve and vascular structures closely intertwined and often separated by a few millimeters. The literature on alternative surgical approaches to the cochlea is extremely limited, while the exact tridimensional topography of the cochlea inside the petrous bone has been scarcely studied. Although variants to CI surgery have been described, challenges pertaining to the anatomy of the site still abound when the classic transmastoid approach cannot be elected.

This study aimed to produce a detailed description of a new approach to cochlear implant surgery via the MCF which allows for the precise location of the basal turn of the cochlea.

## METHOD

This exploratory anatomy study was held at the Surgical Skills in Otorhinolaryngology Lab of the Medical School of the University of São Paulo (FMUSP). It was approved by the Ethics in Research Committee of the FMUSP, under research protocol # 309/11.

Fifty temporal bones of adult cadavers of both genders preserved in formaldehyde were used in this study. The included bone specimens had adequate squamous and petrous portions, as well as the dura mater of the middle cranial fossa.

**Figure 1:**
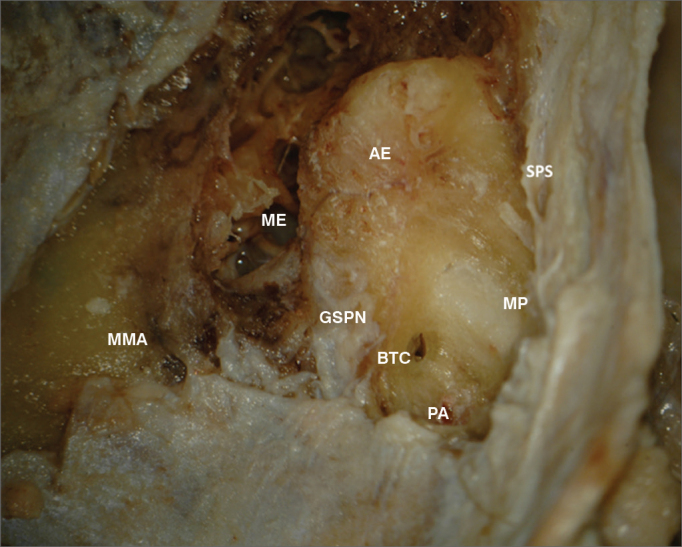
Anatomy of the middle cranial fossa. ME: Middle ear; AE: Arcuate eminence; SPS: Superior petrosal sinus; GSPN: Greater superficial petrosal nerve; MP: Meatal plane; BTC: Basal turn of the cochlea; PA: Petrous apex; MMA: Middle meningeal artery.

**Figure 2:**
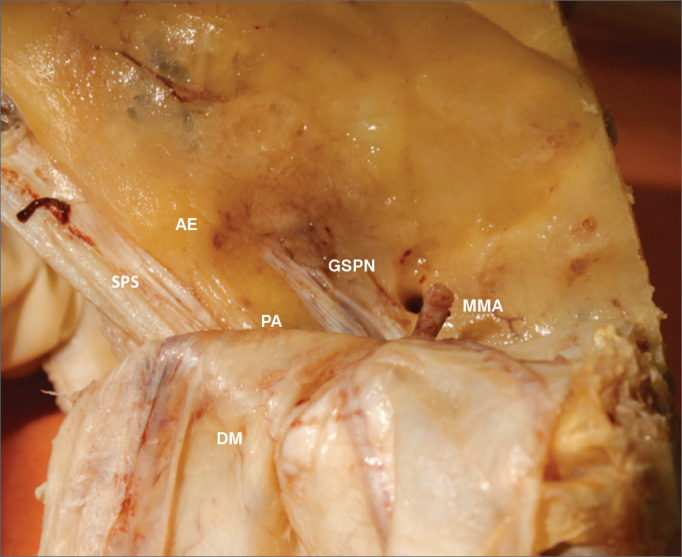
Anatomy of the middle cranial fossa viewed perpendicularly from the petrous. AE: Arcuate eminence; SPS: Superior petrosal sinus; GSPN: Greater superficial petrosal nerve; PA: Petrous apex. DM: Dura mater of the middle cranial fossa; MMA: Middle meningeal artery.

The anatomic landmarks used were the superior petrosal sinus, the stripped petrous apex, the lateral surface of the meatal plane followed on the petrous apex from its more proximal portion (in reference to the projection of the acoustic pore), and the greater superficial petrosal nerve (Figures 1 and 2). The selection of landmarks was carried out based on how easily they could be recognized on the MCF floor and their surgical relevance in locating the basal turn of the cochlea.

The temporal bones were placed in the position in which they would be seen during surgery using the MCF approach. Surgery was performed in accordance with the steps described below:

Exposure of the lateral-superior petrous portion of the temporal bone by detaching the dura mater until the middle meningeal artery was identified.

Visualization of the MCF floor and identification of the greater superficial petrosal nerve, arcuate eminence, and superior petrosal sinus. Medial drilling of the petrous apex toward the meatal plane area, adjacently to the superior petrosal sinus and anteriorly to the acoustic pore.

Identification of the dura mater of the internal acoustic meatus (IAM) by transparency.

Drilling along the greater axis of the IAM until its lateral extremity is identified and, right in front of it, until the more superficial portion of the basal turn of the cochlea is found and opened.

Cochleostomy with a 1 mm diamond tip drill (usually in an area of 2.0 mm in diameter).

Visualization of the osseous spiral lamina separating the scala tympani and scala vestibuli.

Placement of a dummy array through the scala tympani, oriented in the direction of the arcuate eminence.

## RESULTS

The superficial part of the basal turn of the cochlea was easily found through this approach in all 50 temporal bones. The exposure of the basal turn of the cochlea allowed the visualization of the scala tympani and scala vestibuli. Thus, the array could be easily placed through the scala tympani.

**Figure 3:**
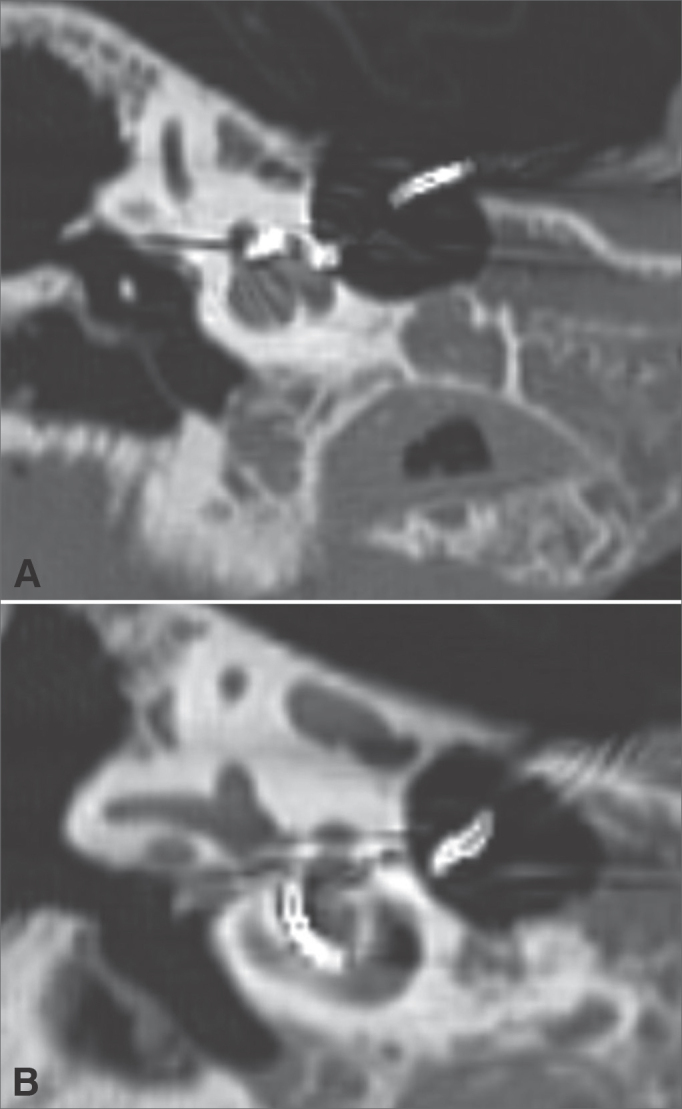
A-B: Right temporal bone high-resolution CT scan. Coronal view, bone window, showing the placement of the array from the basal (A) to the apical (B) turn of the cochlea.

**Figure 4:**
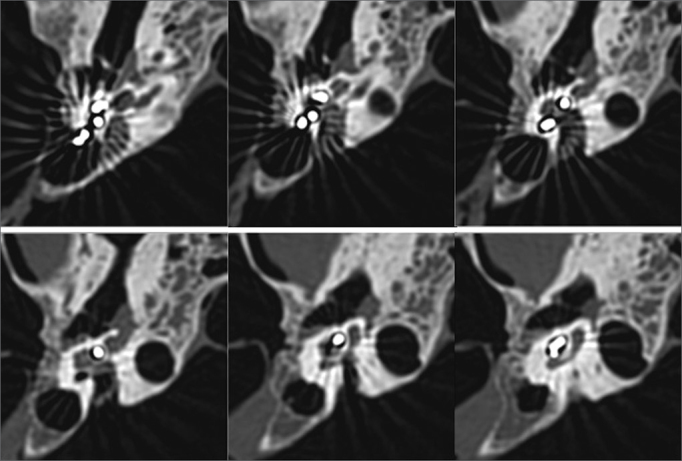
Right temporal bone high-resolution CT scan. Axial view, bone window, showing the placement of the array from the basal to the apical turn of the cochlea.

**Figure 5:**
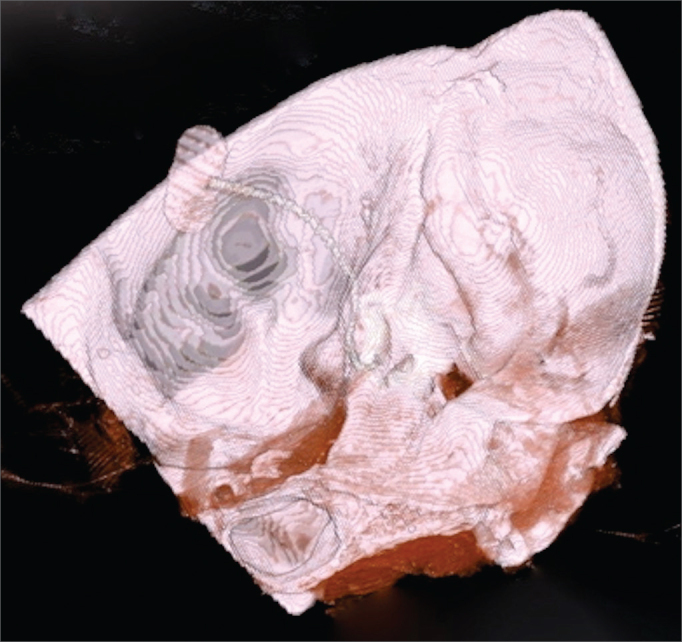
Right temporal bone high-resolution CT scan. 3D reconstruction showing the placement of the CI array through the middle cranial fossa.

The placement of the dummy CI array was documented through temporal bone computerized tomography scans (Figures 3, 4, and 5).

## DISCUSSION

Many authors have reported variations in the anatomy of the MCF likely to be related to differences in aeration of the temporal bone^18^, ^20^, ^22^, ^23^, ^24^, ^25^, ^26^. Various methods to identify the position of the IAM have been described^18^, ^21^, ^27^.

House & Shelton^27^ followed the greater superficial petrosal nerve until the facial nerve, so as to reach the IAM directly. However, this approach requires meticulous technique to avoid damage to the facial nerve.

The meatal plane approach published by Fisch^24^, ^25^ was developed to limit dura mater retraction and mitigate the risk of introducing damage to the labyrinthine segment of the facial nerve. In this approach, the superior semicircular canal is identified by its ‘blue line' (visualization of the membranous labyrinth by transparency), while a 60° angle from the end of its long axis is used to define the safe zone to drill and locate the IAM.

Garcia-Ibañez & Garcia-Ibañez^26^ proposed that the bisection of the angle formed by imaginary lines drawn along the greater superficial petrosal nerve and the arcuate eminence could be used as a reference to reach the IAM. This approach does not require drilling along the superior semicircular canal or the geniculate ganglion, thus minimizing the risk of injuring these structures. However, the arcuate eminence cannot be identified in all cases^18^ or match the exact position of the superior semicircular canal^20^.

Bento et al.^17^ described a quick and safe approach to expose the geniculate ganglion and the labyrinthine portion of the facial nerve through the MCF, exploring the ceiling of the middle ear cavity. This approach includes the identification of the cochleariform process and the opening of the *tegmen tympani*.

Jackler & Gladstone^28^ used a dissection technique starting in the direction of the medial face (anteriorly to the acoustic pore) toward the lateral portion of the petrous apex to identify the IAM.

Few authors have looked into the projections and anatomic relations of the cochlea while approaching the turns of the cochlea^14^, ^23^. The meatal plane has not been mentioned in the literature as a landmark for the basal turn of the cochlea, thus preventing any comparison.

A review published by Colletti et al.^3^, ^2^, ^13^ revealed that 12 patients underwent CI surgery via the MCF approach. The authors stated that this was their approach of choice when treating patients with postoperative mastoid cavities, middle ear chronic disease and malformations, or partial ossification of the basal turn of the cochlea. The superior projection of the basal turn of the cochlea was located on the MCF floor in the angle formed by the greater superficial petrosal and facial nerves, where the cochleostomy was performed and the array placed. However, it is the apical turn of the cochlea that correlates to these structures.

As seen on the CT scans, the array reached almost the entire length of the cochlea, with only a few millimeters remaining between the round window and the cochleostomy. The stimulation of the middle and apical portions of the cochlea by the implant involves more nerve interactions than the stimulation by the array of the basal turn of the cochlea^14^. Thus, we believe that patients offered this approach will not be harmed.

In all 50 temporal bones included in this study, only the superior part of the basal turn of the cochlea was uncovered. Exposure of the basal turn of the cochlea (usually an area of 2.0 mm in diameter) allowed the visualization of the scala tympani and scala vestibuli. The placement of the cochlear implant through the scala tympani oriented in the direction of the arcuate eminence was significantly eased. Even though this study was performed on specimens of temporal bone removed from the skull, the MCF approach has been reproduced in cadavers in surgery-like conditions. A bone window measuring 3 × 4 cm was produced on the squamous part of the temporal bone and the temporal lobe was retracted without leading to any additional difficulty accessing the basal turn of the cochlea or placing the dummy array.

Exposure of part of the petrous apex requires more retraction of the temporal lobe and often calls for obliteration of the middle meningeal artery^29^. However, as drilling is done only adjacently to the lateral face of the IAM, the amount dural retraction will vary depending on the anatomy of the patient's MCF floor.

The approach described in this paper appears to be simpler and more reliable in locating the cochlea. It also ensures sufficient exposure of the basal portion of the cochlea while avoiding injury to other structures. The basal turn of the cochlea is located immediately below the MCF floor and can be easily accessed by drilling the bone lateral to the meatal plane, without posing harm to vital structures, once in this path there is only aerated bone. It is also possible to visualize the osseous spiral lamina and place the CI array through the scala tympani, reaching almost the entire length of the organ of Corti.

## CONCLUSION

The approach described in this paper simplifies the cochleostomy procedure and the placement of the array. When performed through the MCF approach, cochlear implant surgery takes less time, reduces the occurrence of surgical trauma, and mitigates postoperative complications. Additionally, facial nerve damage is avoided, as this approach does not require the stripping of any portion of the facial nerve, as seen in other popular procedures.
